# Complete mitochondrial DNA sequence of the Eastern Asian catfish *Silurus asotus* (Siluriformes: Siluridae) from Lake Biwa in Japan

**DOI:** 10.1080/23802359.2022.2034546

**Published:** 2022-02-07

**Authors:** Yuu Kishimoto, Hisashi Okuyama, Jun-ichi Takahashi

**Affiliations:** Faculty of Life Sciences, Kyoto Sangyo University, Kamigamo, Kyoto, Japan

**Keywords:** Next-generation sequencing, catfish, Lake Biwa, *Silurus asotus*, mitochondrial DNA

## Abstract

The east Asian catfish *Silurus asotus* is a common species living in fresh water in Japan. The complete mitochondrial genome of the *S. asotus* from Lake Biwa in Japan was analyzed using next-generation sequencing. The mitochondrial genome of *S. asotus* was identified as a 16,515 bp circular molecule containing 13 protein-coding genes (PCGs), 22 tRNA genes, and two rRNA genes, along with one A + T-rich control region. The AT content was 56.1%. Start codons ATG and GTG were found in 13 PCGs. Stop codons TAA, TAG, and AGA were observed in 13 PCGs. The heavy (H)-strand was predicted to have 12 PCGs and 14 tRNA and two rRNA genes, while the light (L)-strand was predicted to contain one PCGs and eight tRNA genes. The molecular phylogenetic analysis showed that *S. asotus* from Lake Biwa is genetically similar to *S. asotus* from China.

The *Silurus asotus* is one of four species of the genus *Silurus* distributed in Japan and is a common species with a wide distribution (Nakatani et al. 2011). Lake Biwa in Japan is an ecosystem with many endemic and endangered species due to its high fish biodiversity, and the genus *Silurus* is at the top of the food chain (Kishimoto et al. 2021a, 2021b). Here, we first report the complete mitochondrial genome of the *S. asotus* from Lake Biwa in Japan.

DNA samples from the fin of *S. asotus* found in Lake Biwa of Japan (34°58′N, 135°54′E), were immediately extracted using DNA blood and tissue mini kit (Qiagen, Hilden, Germany). A specimen was deposited at the Shiga Prefectural Lake Biwa Museum, Japan (https://www.biwahaku.jp, R. Tabata and query@biwahaku.jp) under the voucher number LBM1210058080. The gDNA library used for sequencing was prepared using the KAPA Hyper Prep kit, and a MiSeq sequencer (Illumina, San Diego, CA, USA) was used to sequence the whole genome with an Illumina reagent kit. The gDNA library was indexed and run simultaneously over 600 cycles yielding paired reads of 250 bp. Genomic DNA was sequenced using Illumina’s MiSeq platform (Illumina). The resultant reads were assembled with Geneious R9 (Biomatters, Auckland, New Zealand) (Kearse et al. 2012) using *S. asotus* (AP012022) as the reference sequence. The 37 genes were annotated using the MITOS web server (Bernt et al. 2013).

We succeeded in sequencing the entire mitochondrial genome of *S. asotus* from Lake Biwa, Japan. This sequence is DDBJ accession number LC574780. The genome consisted of a closed-loop 16,515 bp long, which included 13 PCGs, 22 tRNA genes, two rRNA genes, and one AT-rich control region. All PCGs began with ATG as the start codon, except *COI*, which had GTG as the start codon. Stop codons were variable for all PCGs: six genes used TAA; and one gene used AGA; and four genes used TAG as the stop codon. The *COIII* and *Cytb* genes have incomplete termination codons ending in U and UA, respectively. Incomplete terminal codons have been identified in some vertebrates including fish (Oh et al. 2007; Zeng et al. 2011; Wang K et al. 2015; Wang QR et al. 2015). Phylogenetic trees based on 13 protein-coding genes of 14 *Silurus* taxa and *Kryptopterus bicirrhis* and *Ompok bimaculatus* as outgroup were constructed using MEGA X (Kumar et al. 2018) with the maximum-likelihood method and the GTR G + I substitution model. The substitution model was selected utilizing the Find Best DNA/protein model tool of MEGA X. The node reliability was assessed by performing 1000 rapid bootstrap replicates.

Phylogenetic analysis suggests that the Lake Biwa *S. asotus* are genetically distinct from the Japanese river *S. asotus* previously analyzed (Figure 1). A new species of the genus *Silurus*, *S. tomodai*, Hibino and Tabata, 2018 was described in Japan (Hibino and Tabata 2018). *S. tomodai* is morphologically similar to *S. asotus*. We consider that the Japanese catfish *S. asotus*, which mitochondrial DNA has been analyzed in the past (Nakatani et al. 2011), needs to be revalidated for species identification.

**Figure 1.**
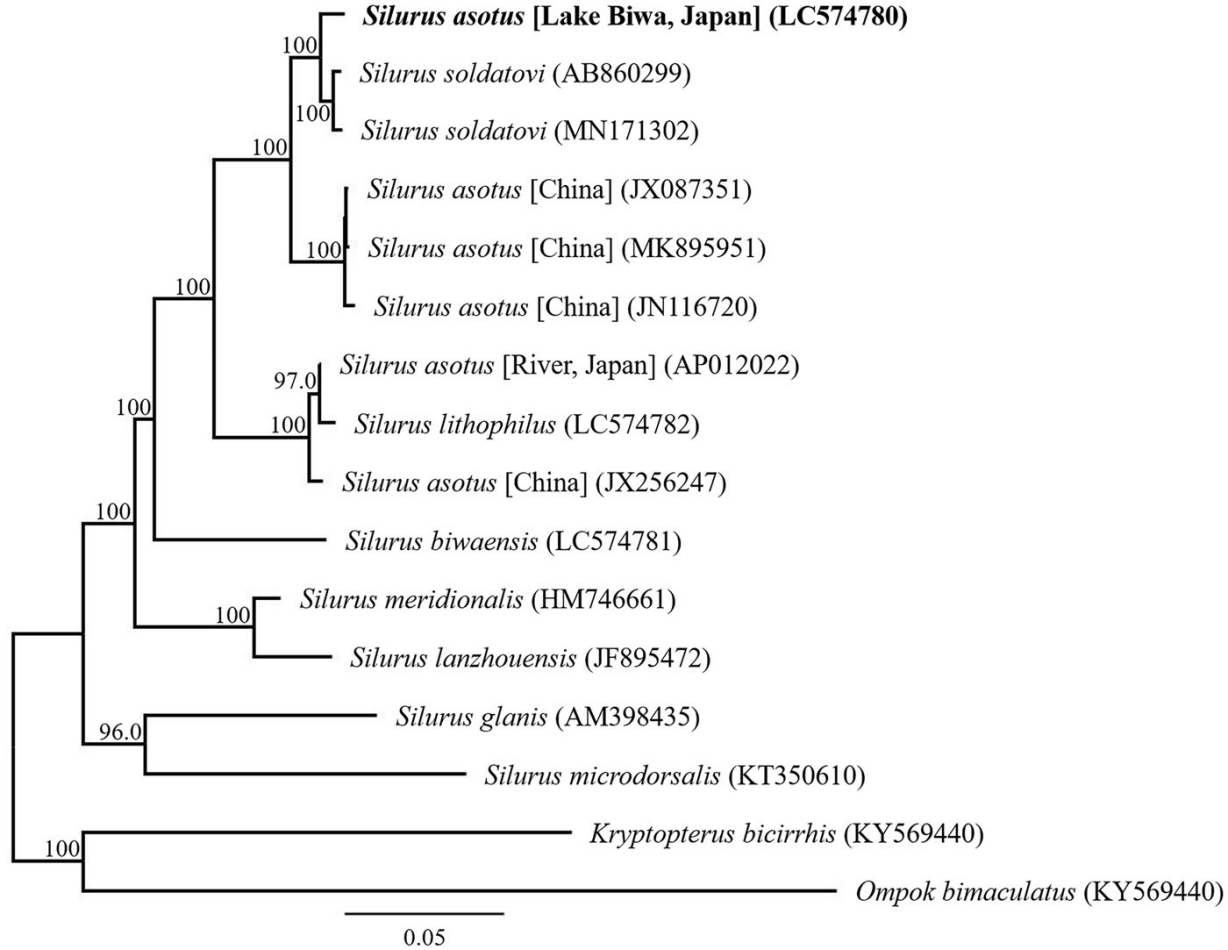
Phylogenetic relationships (maximum likelihood) of the catfish (Siluridae) based on the nucleotide sequences of the 13 protein-coding genes of the mitochondrial genome. The numbers at the nodes indicate the bootstrap support inferred from 1000 bootstrap replicates. Alphanumeric terms indicate the DNA Database of Japan accession numbers. Boldface indicates sample analyzed in this study.

## Data Availability

The genome sequence data that support the findings of this study are openly available in DDBJ/GenBank at https://www.ddbj.nig.ac.jp/indexhtml under accession no. LC574780. The associated BioProject ID, BioSample ID, and SRA (DRA) Accession no. are PRJDB11344, SAMD00283507, and DRA011644, respectively.
